# Post-pancreatic necrosectomy biliary fistula: thinking beyond the box

**DOI:** 10.1093/jscr/rjab189

**Published:** 2021-05-14

**Authors:** Narendra Pandit, Laligen Awale, Kunal Bikram Deo, Bickram Pradhan

**Affiliations:** 1 Division of Surgical Gastroenterology, Department of Surgery, B. P. Koirala Institute of Health Sciences (BPKIHS), Dharan, Nepal; 2 Department of Gastroenterology and Hepatology, B. P. Koirala Institute of Health Sciences (BPKIHS), Dharan, Nepal

## Abstract

Walled-off pancreatic necrosis is a challenging problem and pancreatic necrosectomy is associated with significant morbidity and mortality. Following necrosectomy, postoperative bile leak is a rare complication. We present such a case of delayed bile leak from the distal common bile duct in an 81-year-old lady following pancreatic necrosectomy, which was successfully managed by endoscopic stenting.

## INTRODUCTION

Walled-off pancreatic necrosis can be managed by percutaneous catheter drainage, endoscopic, minimally invasive technique or open necrosectomy [1]. Following all the above procedures, around 10 to 40% of the patients commonly develop enteric fistula, pancreatic fistula and bleeding complications [2, 3]. Apart from these, rarely do the patients develop biliary fistula due to sloughing of the bile duct wall by the necrosis and pancreatic enzymes. Here we discuss such rare event of biliary fistula developing in a delayed fashion following necrosectomy at fourth week of surgery after discharge.

## CASE REPORT

**Figure 1:**
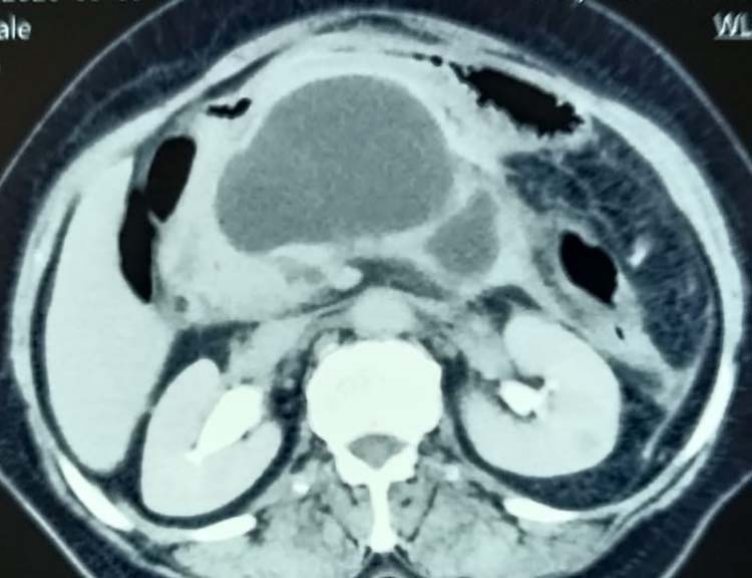
Contrast CT abdomen showing walled-off pancreatic necrosis in the pancreatic head, close to the distal bile duct.

**Figure 2:**
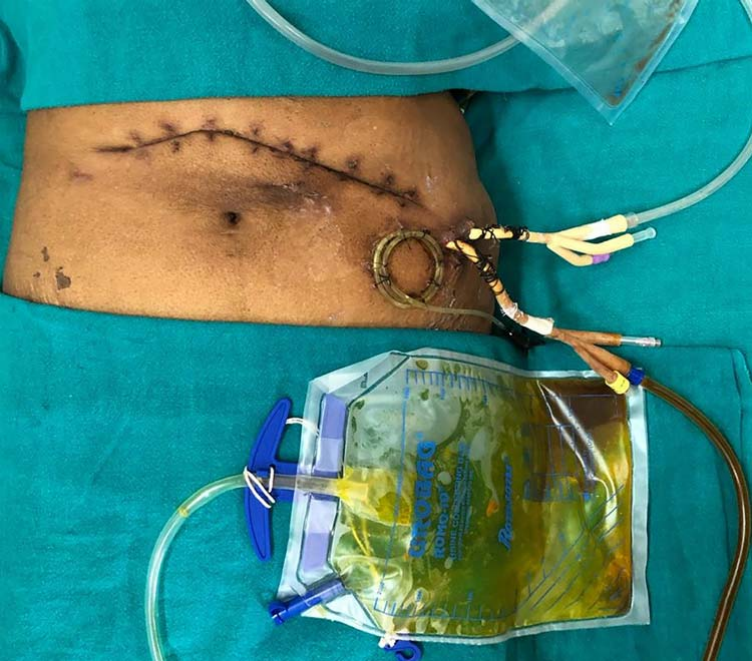
Imaging showing post-pancreatic necrosectomy status with bile leak in the lesser sac drain.

**Figure 3:**
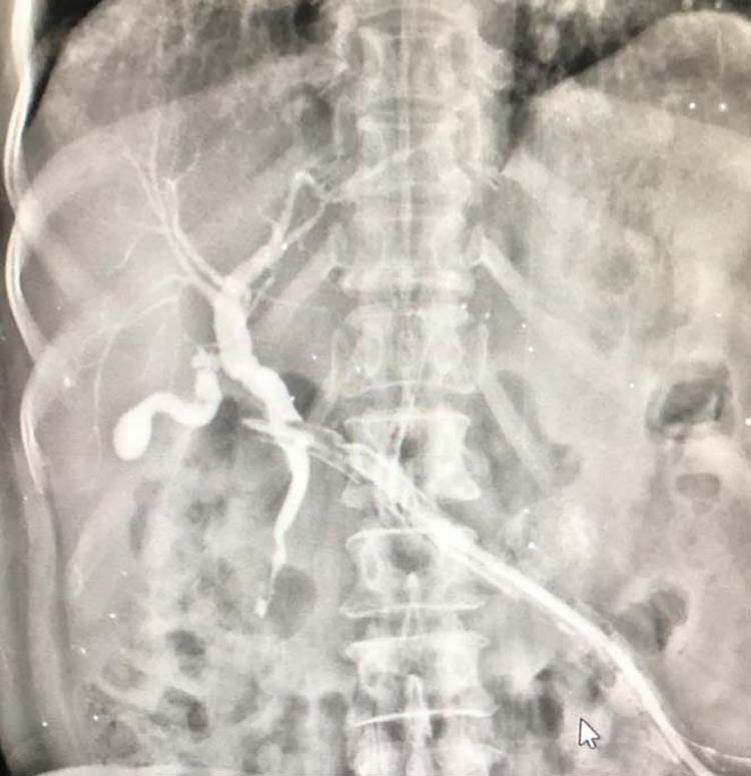
Fistulogram from the lesser sac drain showing biliary tree opacification.

An 81-year-old female, a known case of idiopathic acute necrotizing pancreatitis, had been on percutaneous catheter drainage (PCD) for infected pancreatic necrosis at 6 weeks of onset (Fig. 1). On the eight day, she required a second PCD insertion for persistent lesser sac collection and sepsis. During PCD intervention, there was active bleeding from the catheter site which led to emergency pancreatic necrosectomy, hemostasis from the bleeding gastrocolic ligament vessel, closed lesser sac drainage and feeding jejunostomy. After surgery, she did well and was discharged on Day 15. The lesser sac drain output was clear with a raised amylase level. On the 24th day after surgery, she was readmitted for the high (300 ml/day) bilious drain output (Fig. 2). She denied any fever, vomiting or abdominal pain. She was hemodynamically stable without abdominal distention or tenderness. Her blood investigations revealed leukocytosis (13 600 cells per cubic milliliter), but normal renal function tests and serum chemistry. A diagnosis of the duodenal fistula was presumed and was kept nil per oral, intravenous fluids, antibiotics and initiation of feeding jejunostomy. She was doing well for the next 1 week with a good appetite. Contrast computed tomography (CT) excluded any peripancreatic collections, and importantly there was no oral contrast extravasation in the para-duodenal region. The drain output was persistently high (300–350 ml/day) and bilious with normal fluid amylase activity. We requested for fistulogram, which surprisingly revealed an external biliary fistula from the distal common bile duct (Fig. 3). Endoscopic retrograde cholangiopancreatography (ERCP) and bile duct stenting (7Fr × 10 cm double pigtail plastic stent) was performed, which led to the closure of the fistula over the next 7 days. At a 6-month follow-up, the patient is doing well.

## DISCUSSION

Following severe necrotizing pancreatitis, the most common cause for the bile leak either spontaneously or following surgery is the duodenal fistula [3, 4]. Rarely, it can be due to the erosion of the distal common bile duct by the peri-pancreatic necrosis [5, 6]. Though it presents early following intervention, in the present case the bile leak became obvious only after the third week of necrosectomy after discharge from the hospital. This may be because of the compression and delayed sloughing of the bile duct by the pancreatic enzymes [5]. A high index of suspicion is required for the diagnosis of biliary fistula. The patient is usually well-doing, tolerates oral feed without a leak from the drain, low output with a golden yellow tinge, and normal fluid amylase activity. If evident, it can be successfully managed by ERCP and biliary stenting.

## CONCLUSION

The most common cause of bilious output from the drain following pancreatic necrosectomy is duodenal fistula; however, a high index of suspicion of biliary fistula (though rare) from the bile duct should be made if the patient is well and tolerating feed.

## AUTHOR CONTRIBUTIONS

N.P. wrote the manuscript. L.A., K.B.D. and B.P. provided the images. N.P., L.A., K.B.D. and B.P. approved the final manuscript. N.P. is the article guarantor.

## CONFLICT OF INTEREST STATEMENT

None declared.

## FUNDING

None.

## INFORMED CONSENT

Informed consent was obtained for this case report.
